# FFCA: a feasibility-based method for flux coupling analysis of metabolic networks

**DOI:** 10.1186/1471-2105-12-236

**Published:** 2011-06-15

**Authors:** Laszlo David, Sayed-Amir Marashi, Abdelhalim Larhlimi, Bettina Mieth, Alexander Bockmayr

**Affiliations:** 1 DFG-Research Center Matheon, Berlin, Germany; 2 FB Mathematik und Informatik, Freie Universität Berlin, Arnimallee 6, D-14195 Berlin, Germany; 3 Berlin Mathematical School (BMS), Berlin, Germany; 4 International Max Planck Research School for Computational Biology and Scientific Computing (IMPRS-CBSC), Max Planck Institute for Molecular Genetics, Ihnestr. 63-73, D-14195 Berlin, Germany; 5 Department of Bioinformatics, Institute for Biochemistry and Biology, University of Potsdam, Karl-Liebknecht-Str. 24-25, D-14476 Potsdam, Germany; 6 School of Mathematics, University of Southampton, Highfield, Southampton, SO17 1BJ, UK

## Abstract

**Background:**

Flux coupling analysis (FCA) is a useful method for finding dependencies between fluxes of a metabolic network at steady-state. FCA classifies reactions into subsets (called coupled reaction sets) in which activity of one reaction implies activity of another reaction. Several approaches for FCA have been proposed in the literature.

**Results:**

We introduce a new FCA algorithm, FFCA (Feasibility-based Flux Coupling Analysis), which is based on checking the feasibility of a system of linear inequalities. We show on a set of benchmarks that for genome-scale networks FFCA is faster than other existing FCA methods.

**Conclusions:**

We present FFCA as a new method for flux coupling analysis and prove it to be faster than existing approaches. A corresponding software tool is freely available for non-commercial use at http://www.bioinformatics.org/ffca/.

## Background

Constraint-based analysis of metabolic networks has become increasingly important for describing and predicting the behavior of living organisms [1,2]. While a growing number of metabolic network reconstructions have become available during the last years, the computational analysis of genome-scale networks with hundreds or thousands of reactions may still be very time-consuming. Therefore, there is a need for more efficient algorithms and tools (see e.g. [3-5]).

Flux coupling analysis (FCA) [6] is a useful method for finding dependencies between fluxes of a metabolic network at steady-state. If a non-zero flux through a reaction in steady-state implies a non-zero flux through another reaction, then the first reaction is said to be coupled to the second reaction. Several studies have used FCA for exploring various biological questions such as network evolution [7-9], gene essentiality [7], analysis of experimentally measured fluxes [10,11] or gene regulation [12,13]. Having a time efficient implementation of FCA is important in such studies.

In the rest of this section, we first recall some basic definitions. Then we briefly review the previously proposed FCA methods.

### Mathematical definitions

#### Basic preliminaries

For a metabolic network with *m *(internal) metabolites and *n *reactions, the *stoichiometric matrix S *is an *m *× *n *matrix, where element *S_ij _*is the stoichiometric coefficient of metabolite *i *in reaction *j*. We denote by *R *= {1, ..., *n*} the set of reactions and by M = {1, ..., *m*} the set of metabolites.

There are two types of reactions in a metabolic network. *Irr *is the set of *irreversible *reactions which are assumed to always have non-negative flux values. *Rev *is the set of *reversible *reactions which are allowed to have positive, negative or zero flux values. If a reversible reaction has a positive (resp. negative) flux, we say that it is working in *forward *(resp. *backwar*d) direction. *Splitting *a reversible reaction *i *means making reaction *i *irreversible and adding one more irreversible reaction *i *+ *n *to the network, which works in the backward direction. Without loss of generality, we assume that in the numbering of reactions, the first |*Rev*| reactions are the reversible ones.

If *v *is the vector of metabolic fluxes through all the reactions, the *steady*-*state *condition holds for *v *if *S *· *v *= 0. The steady-state *flux cone *is defined as *C *= {*v *∈ ℝ^*n *^| *S *· *v *= 0, *v_i_ ≥*0, for all *i *∈ *Irr*}. The lineality space of *C *is given by *lin*.*space*(*C*) = {*v *∈ ℝ^*n *^| *S *· *v *= 0, *v_i _*= 0, for all *i *∈ *Irr*}.

If for some reaction *r *∈ *R*, *v_r _*= 0 for all *v *∈ *C*, then *r *is called *blocked*, otherwise *r *is *unblocked*. The set of blocked reactions will be denoted by *Blk*. The set of unblocked reactions can be further partitioned based on the *reversibility type *of reactions [14]. We define *Irev *as the set of all reactions that can work only in one direction, i.e., those reactions that take only non-negative or only non-positive flux values at steady-state. If *i *∈ *Irev *and *v_i _*≤ 0 for all *v *∈ *C*, one can multiply by -1 the *i*-th column of the stoichiometric matrix, such that in the modified network, we have *v_i _*≥ 0 for all *v *∈ *C*. Without loss of generality, we assume that all reactions in *Irev *are operating in the forward direction.

The set of reactions that can work in both directions at steady-state is further partitioned into two subsets: (*a*) *Prev*, the set of *pseudo*-*irreversible *reactions, and (*b*) *Frev*, the set of *fully reversible *reactions. A reaction *i *is in *Prev *if it can work in both directions, and additionally, for all flux vectors *v *in the lineality space of the flux cone, we have *v_i _*= 0. Accordingly, we define *Frev *as the set of those reactions, which can work in both directions, and additionally, can have a non-zero flux value if fluxes through all irreversible reactions are set to zero.

#### Flux coupling relations between a pair of reactions [6]

Consider two unblocked reactions *i*, *j *∈ *R*. If for all *v *∈ *C*, *v_i _*≠ 0 implies *v_j _*≠ 0, then *i *is *directionally coupled *to *j*. This will be denoted by *i *→ *j*, or equivalently, *j *← *i*. If for all *v *∈ *C*, *v_i _*≠ 0 implies *v_j _*≠ 0 and vice versa, then we say that *i *and j are *partially coupled*, or *i *↔ *j*. If *i *and *j *are partially coupled, and additionally there exists a constant *k *such that for all *v *∈ *C*, *v_i _*≠ 0 implies *v_j_*/*v_i _*= *k*, then *i *and *j *are *fully coupled*, or *i *⇔ j. If neither *i *→ *j *nor *j *→ *i *holds, then *i *and *j *are *uncoupled*, or .

#### Elementary flux patterns

The set *supp*(*v*) = {*i *∈ *R *| *v_i _*≠ 0} of reactions active in *v* is called the *support *of *v*. Suppose *v *∈ *C *is a flux vector and *A *⊆ *R *is a subnetwork. The flux pattern of *v *for *A *is defined as *A *∩ *supp*(*v*), which is the set of those reactions in *A* which have non-zero values in *v*[15]. A flux pattern is called an *elementary flux pattern* (EFP) if it cannot be written as the union of other flux patterns. For studying EFPs, Kaleta et al. [15] assume that the network contains only irreversible reactions. To achieve this, every reversible reaction should be split into two irreversible reactions (forward and backward).

### Approaches to Flux Coupling Analysis

In this subsection, we briefly recall existing approaches to flux coupling analysis. For additional information and the technical details on the implementation of these algorithms, we refer to Additional file 1.

#### Flux Coupling Finder Algorithm (FCF)

The most widely used method for FCA is the Flux Coupling Finder (FCF) algorithm [6]. This approach is based on solving linear programs (LPs), and therefore is an optimality-based method. After finding blocked reactions and splitting reversible reactions, for every pair of unblocked reactions *i *and *j*, two LPs are solved. Depending on the optimal values obtained, the coupling relation between *i *and *j *is determined. There is a post-processing step in FCF. Since the reversible reactions have been split, flux coupling relations for these reactions have to be obtained from the coupling relations of the corresponding irreversible forward and backward reactions.

The FCF algorithm has been successfully used for finding coupling relations in a number of metabolic networks [6-13,16]. However, this approach is rather time-consuming for genome-scale metabolic networks with thousands of reactions, although it is still one of the fastest FCA methods.

#### FCA based on Minimal Metabolic Behaviors (MMB-FCA)

Larhlimi and Bockmayr [14,17] have proposed a different strategy for flux coupling analysis. In this approach, a minimal set of generating vectors of the flux cone is computed. Then, the coupling relation for any pair of reactions is inferred based on the co-appearance of non-zero fluxes in the generating vectors.

#### FCA based on elementary flux patterns (EFP-FCA)

Recently, Kaleta et al. [15] introduced the concept of elementary flux patterns (EFPs) for the analysis of minimal active reactions in a "subnetwork", which account for possible steady-state flux distributions in a (much) larger metabolic network. They also presented a method based on mixed-integer linear programming (MILP) to compute EFPs. Kaleta et al. suggested that EFPs can be used for characterizing flux coupling relations (see Supplemental Material in [15]). Consider a subnetwork including two unblocked reactions *i *and *j*. If each of these reactions can have a non-zero flux independently of the other (i.e. ), {*i*} and {*j*} are the only EFPs in this subnetwork. On the other hand, if we assume (without loss of generality) that *i *is directionally coupled to *j*, then the EFPs of this subnetwork are {*i*, *j*} and {*j*}. Finally, if the reactions are partially coupled, we will have only one EFP, which is {*i*, *j*}. With this method, it is not possible to distinguish between partial and full coupling, since flux patterns only contain the information about the activity or inactivity of the fluxes, but not the flux values.

### Further strategies for improving flux coupling analysis

In FCF, and some other FCA approaches, every reversible reaction is split into a forward and a backward reaction. This splitting procedure results in an increase in the number of LPs and also in the size of each LP to be solved. Moreover, a complicated postprocessing step is required to infer the flux coupling relations of the original reversible reactions. For all these reasons, it might be better to perform flux coupling analysis without splitting the reversible reactions. An implementation of FCF without splitting is presented in Additional file 1.

Additionally, Larhlimi and Bockmayr [14] show that depending on the reversibility type of the reactions, only certain flux coupling relations can occur. Two unblocked reactions *i *and *j *can be coupled only if one of the following 4 cases holds (note that initially 3 × 3 = 9 reversibility types for the pair (*i*, *j*) would be possible):

1. *i*, *j *∈ *Irev*: In this case, *i *and *j *can be directionally, partially or fully coupled.

2. *i *∈ *Irev *and *j *∈ *Prev*: The only possibility is *j *→ *i*.

3. *i*, *j *∈ *Prev*: In this case, we can only have *i *⇔ *j*.

4. *i*, *j *∈ *Frev*: In this case, we can only have *i *⇔ *j*.

Therefore, it is enough to determine the reversibility types of *i *and *j*, and then check if the corresponding coupling relation holds. This will be referred to as "Reversibility-Type prunings" (or simply, RT-prunings). RT-prunings were originally used in MMB-FCA as presented in [14].

After RT-prunings, a further improvement can be applied to LP-based FCA methods. Assume that in the stoichiomtric matrix *S*, the columns corresponding to the blocked reactions have been removed. According to Proposition 6.13 in [18], for two reactions *i *and *j *such that *i*, *j *∈ *Prev *or *i*, *j *∈ *Frev*, the following two statements are equivalent:

*• i *⇔ *j*

*• *For all *v *such that *Sv *= 0, *v_i _*= 0 implies *v_j _*= 0.

This means that it is sufficient to solve only one LP:

The two reactions *i *and *j *will be fully coupled if and only if in the above LP . Note that the irreversibility constraints are not included in the above LP. Therefore, not only solving one LP is sufficient, but also the LP becomes smaller. This improvement will be called *Prev*/*Frev*-based improvement (or simply, PF-improvement).

An improved version of FCF, which takes into account all of the above-mentioned improvements, has been suggested in [18]. Here we implemented this improved version of FCF and compared it with the other FCA approaches (see the Results and Discussion section).

### Goals of the present work

FCA is a promising tool for metabolic network analysis. However, to perform FCA most authors seem to use their personal implementation of the FCF algorithm [9,13]. Only very recently, an implementation of FCF has been included in the latest versions of the FASIMU software [19,20]. On the other hand, several approaches for FCA have been proposed in the literature. It is not known which of these methods is the fastest in practice. In this paper, we present a novel "feasibility-based" flux coupling analysis method (FFCA) and compare it to previously existing approaches. A corresponding software tool is freely available for non-commercial use.

## Results and Discussion

### FFCA: Feasibility-based Flux Coupling Analysis

A linear programming problem *maximize*(*c^T ^x *| *Ax *≤ *b*) can be solved by enumerating a sequence of feasible solutions, i.e., solutions of the system of linear inequalities *Ax *≤ *b *such that at each solution *x *the objective function value *c^T ^x *improves. Accordingly, finding one feasible solution could be faster than computing an optimal solution (see [21] for the theoretical background).

In this section, we introduce a new approach for flux coupling analysis, FFCA, which is based on feasibility testing. Taking into account the previously mentioned strategies for improving flux coupling analysis, we propose the following procedure for FFCA:

1. *i*, *j *∈ *Irev*: In this case, we check the feasibility of two systems of linear inequalities:(P1)

and(P2)

If (P1) and (P2) are both feasible, then *i *and *j *are not coupled to each other . If (P1) (resp. (P2)) is infeasible, then *i *→ *j *(resp. *j *→ *i*). If (P1) and (P2) are both infeasible, then *i *and *j *are partially (and maybe fully) coupled. To check whether they are fully coupled, one has to use other methods, e.g. computing enzyme subsets [22] or solving two LPs as in the FCF algorithm [6].

2. *i *∈ *Irev *and *j *∈ *Prev*: The only possible coupling relation is *j *→ *i *(but not *i *→ *j*). Hence, (P1) will always be feasible, because feasibility of (P1) means that *i *→ *j *does not hold. However, we need to check the feasibility of (P2). Additionally, since *j *can take negative values, one more system should be checked for feasibility:(P3)

It can be easily shown that if (P2) and (P3) are both infeasible, then *j *→ *i*. Otherwise, *i *and *j *are uncoupled.

3. *i*, *j *∈ *Prev *or *i*, *j *∈ *Frev*: In this case, the following system of linear inequalities should be checked for feasibility:(P4)

If (P4) is infeasible, then *i *⇔ *j *(because feasibility of (P4) implies *j *→ *i*, which in turn implies *i *⇔ *j *based on Proposition 2 in [14]). If (P4) is feasible, then *i *and *j *are uncoupled.

To perform FFCA, a method is needed to check the feasibility of a system of linear inequalities. In practice this can be done by solving an LP constructed by the system of inequalities together with a constant objective function. Any feasible solution will be an optimal solution of the LP, and therefore, the LP solver will finish after finding the first feasible solution. For example, for checking the feasibility of (P1), one can solve the following LP:

Since *v_i _*is constant, the optimal value exists if and only if this problem is feasible. Similar LPs can be solved to check the feasibility of (P2) and (P3).

In Table 1, the characteristics of the FFCA approach are compared to the other FCA methods studied in this article. Note that in all methods, blocked reactions are found and the possible irreversibility of initially reversible reactions is detected within the preprocessing step.

**Table 1:** General comparison of different approaches to flux coupling analysis

Method name	Preprocessing	Type of linear program and solution	Further distinguishing of partial and full coupling required?	Postprocessing for reversible reactions?
MMB-FCA	computing MMBs + reaction classification	n/a	No	No
EFP-FCA	splitting reversible reactions	MILP, optimal	Yes	Yes
FCF	splitting reversible reactions	LP, optimal	No	Yes
W-FCF	n/a	LP, optimal	No	No
WR-FCF/WRP-FCF	reaction classification	LP, optimal	No	No
FFCA	reaction classification	LP, feasible	Yes	No

### Comparison of the four FCA approaches

To compare the different approaches, namely FCF, MMB-FCA, EFP-FCA and FFCA, we implemented all of them in Matlab (see Additional file 1). A benchmark set of six metabolic network models was used for the evaluation. The number of unblocked reactions in these models ranges from 18 to 765. Table 2 summarizes the running times, while Table 3 reports on the resulting coupling relations. One can see that in all cases FFCA is 2 to 3 times faster than FCF and orders of magnitude faster than EFP-FCA. Table 2 also shows that FFCA is more appropriate for FCA in genome-scale networks. MMB-FCA is the fastest method for the three smallest networks. However, for the middle-sized *H. pylori *network and especially for the large networks of *S. cerevisiae *and *E. coli*, FFCA proves to be faster than MMB-FCA. The computation time required for MMB-FCA rapidly grows when the number of reactions increases. This is due to the possibly exponential size of the set of generating vectors which has to be computed before finding the coupled reactions (see Section 4.4 in [18]). EFP-FCA, which is based on solving mixed-integer linear programs, turns out to be much slower than other methods. Although the concept of elementary flux patterns is very useful in the analysis of subnetworks, its applicability in full FCA seems to be limited. 

Both the FCF algorithm and the current implementation of FFCA solve LPs for flux coupling analysis. One might ask why FFCA is faster than the classical FCF method. There are at least five major differences between FCF and FFCA:

**Table 2:** CPU running time (in seconds) required for flux coupling analysis of the benchmark networks when CLP [32] is used as the LP solver

	no. of unblocked reactions	MMB-FCA	EFP-FCA	FCF	W-FCF	WR-FCF	WRP-FCF	FFCA
*ILLUSNET*	18	0.01	26.3	0.25	0.14	0.09	0.06	0.06
*RBC*	38	0.05	152	1.39	0.80	0.68	0.64	0.62
*EC core*	63	0.22	585	6.58	3.03	3.13	2.19	2.15
*H. pylori*	217	69.8	>1 day	196	83.6	67.0	63.3	60.7
*S. cerevisiae*	639	>1 day	>1 day	8.5 × 10^3^	4.0 × 10^3^	3.4 × 10^3^	3.3 × 10^3^	3.1 × 10^3^
*E. coli *(iJR904)	765	>1 day	>1 day	1.2 × 10^4^	7.4 × 10^3^	6.3 × 10^3^	5.9 × 10^3^	5.6 × 10^3^

**Table 3:** Number of flux (un-)coupling relations for each of the benchmark networks (Table 2)

	no. of unblocked reactions	total no. of reactions	fully coupled	partially coupled	directionally coupled	uncoupled
*ILLUSNET*	18	19	6	0	9	138
*RBC*	38	42	21	0	15	825
*EC core*	63	63	19	0	9	1925
*H. pylori*	217	480	164	3	309	22960
*S. cerevisiae*	639	1144	692	90	2214	200845
*E. coli* (iJR904)	765	930	2567	68	6208	283387

• When an LP is solved in FFCA, finding the first feasible solution is sufficient, while the LPs should be solved to optimality in case of the FCF algorithm.

• In the FCF method, in contrast to FFCA, every reversible reaction is split into two (forward and backward) irreversible reactions. This step slows down the procedure and increases the size of the LPs to be solved.

• For computing the flux coupling relation between any pair of reactions, we always need two LPs in FFCA, while in FCF sometimes more LPs have to be solved. For example, for computing the coupling relation between an irreversible and a reversible reaction (after splitting), four LPs are solved (see Additional file 1).

• The Reversibility-Type prunings [14] to reduce the number of possible coupled reaction pairs are only considered in FFCA.

• The PF-improvements are only considered in FFCA.

One can think of implementing FCF without splitting reversible reactions and/or with the RT-prunings and/or PF-improvement. In order to assess the importance of these issues, three improved versions of FCF were implemented (see the Additional file 1 and also Table 1): (*i*) FCF was re-implemented without splitting reactions (W-FCF); (*ii*) FCF was re-implemented without splitting reactions and with the RT-prunings (WR-FCF); and (*iii*) FCF was re-implemented without splitting reactions and with the RT-prunings and the PF-improvement (WRP-FCF), as suggested in [18].

In Table 2 the computational running times of these methods are also shown. As expected, the three versions of the improved FCF algorithm are better than the classical FCF algorithm, while they are still slower that FFCA.

#### Computational Properties of FFCA

Flux coupling analysis of genome-scale networks can be very time consuming. Only recently (see e.g. [9]) FCA has been used for some of the new genome-scale metabolic networks that contain more than 2000 reactions. To further illustrate our approach, we have applied FFCA to three of these very large networks. The results are reported in Table 4. We can see that FFCA needs 37-46 hours for each of the networks to perform a complete FCA. The numbers of the resulting flux coupling relations are also given in the table.

**Table 4:** Flux coupling analysis of new generation genome-scale metabolic networks

	no. of unblocked reactions	total no. of reactions	fully coupled	partially coupled	directionally coupled	uncoupled	running time (sec)
*H. sapiens* (Recon 1)	2018	3284	1555	4	3503	2030091	1.59 × 10^5^
*E. coli* (iAF1260)	1878	2075	2563	143	12791	1747006	1.66 × 10^5^
*Human hepatocyte *(HepatoNet1)	2309	2309	1463	201	1593	2661329	1.35 × 10^5^

Since FFCA includes the RT-prunings and PF-improvement, it might be interesting to see how the number of coupling relations (and also the running time of FFCA) depends on the number of reversible reactions in a network. This problem is studied in Additional file 2. Briefly, the flux coupling relations have been computed for the original metabolic network of *E. coli *[23], for the modified metabolic network of *E. coli *[24] (which includes a smaller number of irreversible reactions), and for some random networks in between. As expected, the numbers of uncoupled pairs increase (and the running times generally decrease) with the increase in the number of reversible reactions.

## Conclusions

We introduced FFCA as a new method for flux coupling analysis, and proved it to be faster than any other available approach. Our implementation of FFCA is fast enough to perform FCA for every pair of reactions in *S. cerevisiae *and *E. coli *genome-scale networks in a few hours. A corresponding software tool is available for non-commercial use at http://www.bioinformatics.org/ffca/ and we recommend it for FCA of genome-scale networks.

## Methods

### Metabolic network models

Nine metabolic networks were used in this study: (i) *ILLUSNET *network from [14]; (ii) *RBC *: metabolic network of red blood cell [25]; (iii) *EC core*: central metabolic network of *E. coli *[26]; (iv) *H. pylori *genome-scale metabolic network [27]; (v) *yeast *(*S. cerevisiae*) genome-scale metabolic network [28]; (vi) the 2003 genome-scale metabolic network of *E. coli *(iJR904) [23]; (vii) the 2007 genome-scale metabolic network of *E. coli *(iAF1260) [29]; (viii) the metabolic network of human (Recon 1) [30]; and (ix) the metabolic network of human hepatocyte (HepatoNet1) [31].

For FCA, all uptake reactions were assumed to be able to carry non-zero fluxes.

### Implementation of different FCA methods

Unless indicated otherwise, all tools were implemented in Matlab v7.4. Details about the implementation of the different FCA approaches, together with the corresponding pseudo-codes are presented in Additional file 1.

### Comparison of different FCA methods

All computations were performed on a 64-bit Debian Linux system with Intel Xeon 3.0 GHz processor. The running times include the CPU time for preprocessing, computation of flux coupling relations, and post-processing (where necessary).

## List of abbreviations

FCA: Flux Coupling Analysis; FFCA: Feasibility-based Flux Coupling Analysis; FCF: Flux Coupling Finder; MMB-FCA: Flux Coupling Analysis based on Minimal Metabolic Behaviors; EFP-FCA: Flux Coupling Analysis based on Elementary Flux Patterns; LP: Linear Program; MILP: Mixed-Integer Linear Program; RT-prunings: Reversibility-Type prunings; PF-improvement: *Prev*/*Frev*-based improvement.

## Competing interests

The authors declare that they have no competing interests.

## Authors' contributions

SAM, LD and AB presented the original idea of the work and designed the study. AL and LD introduced the improved versions of FCF. BM and LD implemented the tools and carried out the experiments. The manuscript was first drafted by SAM. The final manuscript was approved by all authors.

## Supplementary Material

Additional file 1 **Different approaches to flux coupling analysis and implementation details**. In this file, pseudocodes and implementation details of different FCA methods are presented. Click here for file

Additional file 2 **Dependence of flux coupling analysis on the number of reversible reactions**. In this file, we show how flux coupling relations depend on the number of reversible reactions in the *E. coli *metabolic network. Click here for file

## References

[B1] Reed JL Descriptive and predictive applications of constraint-based metabolic models Proceedings of the 31st Annual International Conference of the IEEE Engineering in Medicine and Biology Society (EMBC 2009) 2009 2009 5460 5463 10.1109/IEMBS.2009.5334064 19964681

[B2] Fell DA Poolman MG Gevorgyan A Building and analysing genome-scale metabolic models Biochemical Society Transactions 2010 38 1197 1201 10.1042/BST0381197 20863283

[B3] Terzer M Stelling J Large-scale computation of elementary flux modes with bit pattern trees Bioinformatics 2008 24 2229 2235 10.1093/bioinformatics/btn401 18676417

[B4] Haus UU Klamt S Stephen T Computing knock-out strategies in metabolic networks Journal of Computational Biology 2008 15 259 268 10.1089/cmb.2007.0229 18331197

[B5] Gudmundsson S Thiele I Computationally efficient flux variability analysis BMC Bioinformatics 2010 11 489 10.1186/1471-2105-11-489 20920235 PMC2963619

[B6] Burgard AP Nikolaev EV Schilling CH Maranas CD Flux coupling analysis of genome-scale metabolic network reconstructions Genome Research 2004 14 301 312 10.1101/gr.1926504 14718379 PMC327106

[B7] Notebaart RA Kensche PR Huynen MA Dutilh BE Asymmetric relationships between proteins shape genome evolution Genome Biology 2009 10 R19 10.1186/gb-2009-10-2-r19 19216750 PMC2688278

[B8] Pál C Papp B Lercher MJ Adaptive evolution of bacterial metabolic networks by horizontal gene transfer Nature Genetics 2005 37 1372 1375 10.1038/ng1686 16311593

[B9] Yizhak K Tuller T Papp B Ruppin E Metabolic modeling of endosymbiont genome reduction on a temporal scale Molecular Systems Biology 2011 7 479 21451589 10.1038/msb.2011.11 PMC3094061

[B10] Suthers PF Chang YJ Maranas CD Improved computational performance of MFA using elementary metabolite units and flux coupling Metabolic Engineering 2010 12 123 128 10.1016/j.ymben.2009.10.002 19837183

[B11] Bundy JG Papp B Harmston R Browne RA Clayson EM Burton N Reece RJ Oliver SG Brindle KM Evaluation of predicted network modules in yeast metabolism using NMR-based metabolite profiling Genome Research 2007 17 510 519 10.1101/gr.5662207 17339370 PMC1832098

[B12] Notebaart RA Teusink B Siezen RJ Papp B Co-regulation of metabolic genes is better explained by flux coupling than by network distance PLoS Computational Biology 2008 4 e26 10.1371/journal.pcbi.0040026 18225949 PMC2211535

[B13] Montagud A Zelezniak A Navarro E de Córdoba PF Urchueguía JF Patil KR Flux coupling and transcriptional regulation within the metabolic network of the photosynthetic bacterium Synechocystis sp. PCC6803 Biotechnology Journal 2011 6 330 342 10.1002/biot.201000109 21226012

[B14] Larhlimi A Bockmayr A A new approach to flux coupling analysis of metabolic networks Computational Life Sciences II, Second International Symposium (CompLife 2006), Cambridge, UK, Volume 4216 of Lecture Notes in Computer Science 2006 205 215

[B15] Kaleta C de Figueiredo LF Schuster S Can the whole be less than the sum of its parts? Pathway analysis in genome-scale metabolic networks using elementary flux patterns Genome Research 2009 19 1872 1883 10.1101/gr.090639.108 19541909 PMC2765277

[B16] Seshasayee ASN Fraser GM Babu MM Luscombe NM Principles of transcriptional regulation and evolution of the metabolic system in E. coli Genome Research 2009 19 79 91 18836036 10.1101/gr.079715.108 PMC2612968

[B17] Larhlimi A Bockmayr A A new constraint-based description of the steady-state flux cone of metabolic networks Discrete Applied Mathematics 2009 157 2257 2266 10.1016/j.dam.2008.06.039

[B18] Larhlimi A New concepts and tools in constraint-based analysis of metabolic networks PhD thesis 2008 Freie Universität Berlin http://www.diss.fu-berlin.de/diss/receive/FUDISS_thesis_000000009198

[B19] Hoppe A Hoffmann S Gerasch A Gille C Holzhütter HG FASIMU: flexible software for flux-balance computation series in large metabolic networks BMC Bioinformatics 2011 12 28 10.1186/1471-2105-12-28 21255455 PMC3038154

[B20] Hoppe A FASIMU, for flux-balance computation in metabolic networks http://www.bioinformatics.org/fasimu/10.1186/1471-2105-12-28 PMC3038154 21255455

[B21] Schrijver A Theory of Linear and Integer Programming 1986 New York: Wiley

[B22] Pfeiffer T Sánchez-Valdenebro I Nuño JC Montero F Schuster S METATOOL: for studying metabolic networks Bioinformatics 1999 15 251 257 10.1093/bioinformatics/15.3.251 10222413

[B23] Reed JL Vo TD Schilling CH Palsson BO An expanded genome-scale model of Escherichia coli K-12 (iJR904 GSM/GPR) Genome Biology 2003 4 R54 10.1186/gb-2003-4-9-r54 12952533 PMC193654

[B24] Kümmel A Panke S Heinemann M Systematic assignment of thermodynamic constraints in metabolic network models BMC Bioinformatics 2006 7 512 10.1186/1471-2105-7-512 17123434 PMC1664590

[B25] Wiback SJ Palsson BO Extreme pathway analysis of human red blood cell metabolism Biophysical Journal 2002 83 808 818 10.1016/S0006-3495(02)75210-7 12124266 PMC1302188

[B26] Palsson BO Systems Biology: Properties of Reconstructed Networks 2006 New York: Cambridge University Press

[B27] Thiele I Vo TD Price ND Palsson BO Expanded metabolic reconstruction of Helicobacter pylori (iIT341 GSM/GPR): an in silico genome-scale characterization of single- and double-deletion mutants Journal of Bacteriology 2005 187 5818 5830 10.1128/JB.187.16.5818-5830.2005 16077130 PMC1196094

[B28] Duarte NC Herrgård MJ Palsson BO Reconstruction and validation of Saccharomyces cerevisiae iND750, a fully compartmentalized genome-scale metabolic model Genome Research 2004 14 1298 1309 10.1101/gr.2250904 15197165 PMC442145

[B29] Feist AM Henry CS Reed JL Krummenacker M Joyce AR Karp PD Broadbelt LJ Hatzimanikatis V Palsson BO A genome-scale metabolic reconstruction for Escherichia coli K-12 MG1655 that accounts for 1260 ORFs and thermodynamic information Molecular Systems Biology 2007 3 121 17593909 10.1038/msb4100155 PMC1911197

[B30] Duarte NC Becker SA Jamshidi N Thiele I Mo ML Vo TD Srivas R Palsson BO Global reconstruction of the human metabolic network based on genomic and bibliomic data Proceedings of the National Academy of Sciences of the United States of America 2007 104 1777 1782 10.1073/pnas.0610772104 17267599 PMC1794290

[B31] Gille C Bölling C Hoppe A Bulik S Hoffmann S Hübner K Karlstädt A Ganeshan R König M Rother K Weidlich M Behre J Holzhütter HG HepatoNet1: a comprehensive metabolic reconstruction of the human hepatocyte for the analysis of liver physiology Molecular Systems Biology 2010 6 411 20823849 10.1038/msb.2010.62 PMC2964118

[B32] Lougee-Heimer R The Common Optimization INterface for Operations Research: Promoting open-source software in the operations research community IBM Journal of Research and Development 2003 47 57 66

