# Supplementary material for: FFCA: a feasibility-based method for flux coupling analysis of metabolic networks
Source: BMC Bioinformatics. 2011 Jun 15;12:236. doi: 10.1186/1471-2105-12-236 (PMC3144024; doi:10.1186/1471-2105-12-236)
Supplement: Additional file 1 — Different approaches to flux coupling analysis and implementation details. In this file, pseudocodes and implementation details of different FCA methods are presented. [file 1471-2105-12-236-S1.PDF]

Additional file 1:

Different approaches to flux coupling analysis and implementation details

Laszlo David, Sayed-Amir Marashi, Abdelhalim Larhlimi, Bettina Mieth and Alexander Bockmayr

In this study, we implemented the different approaches to compare their time efficiency. MMB-FCA, EFP-FCA and FCF are the previous approaches which were (re-)implemented in this study. Additionally, our new method called FFCA, together with three improved versions of the FCF method, namely W-FCF (FCF without splitting reversible reactions), WR-FCF (FCF without splitting reversible reactions and with Reversibility-Type prunings) and WRP-FCF (FCF without splitting reversible reactions, with Reversibility-Type prunings and $Prev/Frev$-based improvement) were implemented in order to get a better picture about the efficiency of the different approaches.

Some definitions

In this text we will use the following notation:

- $[k]$ is used as an abbreviation for the set $\{1, \ldots, k\}$.
- If S is an $m \times n$ matrix, $A \subseteq [m]$ and $B \subseteq [n]$, then $S_{A,B}$ is the submatrix of S formed by the rows in A and the columns in B.

Implementation of MMB-FCA

In this approach, a convex basis of the flux cone is needed. We use the software *cdd* [1], a tool based on the double description method, to compute a minimum set of generating vectors. These correspond to the lineality space and the minimal proper faces (or minimal metabolic behaviors, MMBs) of the flux cone (see [2] for more details). Next the reversibility type of the reactions is determined by computing the sets $Blk, Irev, Prev$, and $Frev$. The flux coupling relations are then obtained as described in [3]. The following pseudo-code (Algorithm 1) summarizes the procedure. $CouplingRelation$ is a matrix where the entry (i, j), with $i < j$, describes the coupling relation between two unblocked reactions i and j.

Algorithm 1: MMB-FCA, an FCA approach based on the generators of flux cone [3]

Input:
- S (the $m \times n$ stoichiometric matrix)
- $Irr \subseteq \{1, \dots, n\}$ (the set of irreversible reactions)

Output:
- Blk (the set of blocked reactions)
- $CouplingRelation$

Initialization:
$Rev := \{1, \dots, n\} \setminus Irr$;
$Blk := \emptyset; Irev := \emptyset; Prev := \emptyset; Frev := \emptyset$;

Preprocessing:
```
/* Classification of reactions based on their reversibility type */
```
$B := \textsc{ComputeLinealitySpace}(S \cdot v = 0, v_i \geq 0, \text{ for all } i \in Irr)$;
$b := \textsc{NumberOfRows}(B)$;
$G := \textsc{ComputeMinimalProperFaces}(S \cdot v = 0, v_i \geq 0, \text{ for all } i \in Irr)$;
$g := \textsc{NumberOfRows}(G)$;
for $i \in R$ **do**
  if $B_{[b],\{i\}} \neq 0$ **then**
    $Frev := Frev \cup \{i\}$
  else if $G_{[g],\{i\}} = 0$ **then**
    $Blk := Blk \cup \{i\}$
  else if $G_{[g],\{i\}} \geq 0$ *or* $G_{[g],\{i\}} \leq 0$ **then**
    $Irev := Irev \cup \{i\}$
  else
    $Prev := Prev \cup \{i\}$
  end
end

Main procedure:
foreach $i, j \in Prev$ *with* $i < j$ **do**
  if $\exists \lambda \in \mathbb{R}$ *such that* $g_i = \lambda g_j$ *for all* $g \in G$ **then**
    $CouplingRelation[i, j] :=$ "$\Longleftrightarrow$";
  else
    $CouplingRelation[i, j] :=$ "$\overset{Un}{\longleftrightarrow}$";
  end
end
foreach $i, j \in Frev$ *with* $i < j$ **do**
  if $\exists \lambda \in \mathbb{R}$ *such that* $g_i = \lambda g_j$ *for all* $g \in G$ *and* $b_i = \lambda b_j$ *for all* $b \in B$ **then**
    $CouplingRelation[i, j] :=$ "$\Longleftrightarrow$";
  else
    $CouplingRelation[i, j] :=$ "$\overset{Un}{\longleftrightarrow}$";
  end
end

(continued on next page)

Algorithm 1: MMB-FCA, an FCA approach based on the generators of flux cone, continued

```
foreach i, j ∈ Irev ∪ Prev with i ≠ j and {i, j} ⊈ Prev do
    if g_i = 0 or g_j ≠ 0 for all g ∈ G then
        CouplingRelation[i, j] := "⟶";
    else
        CouplingRelation[i, j] := "<-Un->";
    end
end
foreach i, j ∈ Irev with i < j do
    if CouplingRelation[i, j] = "⟶" and CouplingRelation(j, i) = "⟶" then
        if ∃λ ∈ ℝ such that g_i = λg_j for all g ∈ G then
            CouplingRelation[i, j] := "⟺";
        else
            CouplingRelation[i, j] := "⟷";
        end
    end
end
```

Implementation of EFP-FCA

We first introduce some auxiliary functions. *minimize* and *maximize* are two functionals used to solve a linear program (LP), i.e., to optimize a linear objective function subject to a set of linear constraints. For example, if $Con = \{Ax \leq b, Cx = d\}$ and c defines the objective function, then $maximize(c^T x \mid Con)$ computes the maximum value of $c^T x$ subject to the constraints Con. If Con admits no real solution, then both *minimize* and *maximize* return the special value $\perp$ (i.e. infeasible). If Con is feasible, but the optimum is unbounded, then *minimize* (resp. *maximize*) return $-\infty$ (resp. $+\infty$).

Any available LP solver can be used to perform these computations. Here, we used CLP, the LP solver from the COIN-OR package [4].

Another function is CouplingRelationRecompute. In some of the FCA approaches, it is necessary to split reversible reactions i in two irreversible reactions, namely i^+ (forward direction) and i^- (backward direction). Suppose that for an irreversible reaction j, we have ⓐ $= CouplingRelation(i^+, j)$ and ⓑ $= CouplingRelation(i^-, j)$. Then a function is needed to compute the flux coupling relation between i and j, based on ⓐ and ⓑ. The aim of CouplingRelationRecompute is to take such an ⓐ, ⓑ pair and compute the flux coupling relation for the original (non-split) reversible reaction. For computing the coupling relation of two reversible reactions after splitting, this function should be called three times, since $CouplingRelation[i, j]$=CouplingRelationRecompute($CouplingRelation(i^+, j)$,$CouplingRelation(i^-, j)$).

Algorithm 2: Procedure COUPLINGRELATIONRECOMPUTE

Input:
- ⓐ $\in \{\longleftarrow, \longrightarrow, \longleftrightarrow, \Longleftrightarrow, \overset{Un}{\longleftrightarrow}\}$ (the first coupling relation)
- ⓑ $\in \{\longleftarrow, \longrightarrow, \longleftrightarrow, \Longleftrightarrow, \overset{Un}{\longleftrightarrow}\}$ (the second coupling relation)

Output: ⓝ $\in \{\longleftarrow, \longrightarrow, \longleftrightarrow, \Longleftrightarrow, \overset{Un}{\longleftrightarrow}\}$ (a new coupling relation)

```
switch (ⓐ, ⓑ) do
    case ("⟺", "⟷")
        ⓝ := "⟷";
    case ("⟺", "⟶")
        ⓝ := "⟶";
    case ("⟺", "⟵")
        ⓝ := "⟵";
    case ("⟺", "⟷(Un)")
        ⓝ := "⟷(Un)";
    case ("⟷", "⟶")
        ⓝ := "⟶";
    case ("⟷", "⟵")
        ⓝ := "⟵";
    case ("⟷", "⟷(Un)")
        ⓝ := "⟷(Un)";
    case ("⟶", "⟵")
        ⓝ := "⟷(Un)";
    case ("⟶", "⟷(Un)")
        ⓝ := "⟷(Un)";
    case ("⟵", "⟶")
        ⓝ := "⟷(Un)";
    case ("⟵", "⟷(Un)")
        ⓝ := "⟷(Un)";
    otherwise
        ⓝ := ⓐ;
    end
end
```

We also introduce the function BLKFINDERREVCORRECTOR(S, Irr) to find the blocked reactions and correct the reversiblity types of those reactions which can work either in forward or in backward direction. Algorithm 3 summarizes this procedure.

Algorithm 3: Procedure BlkFinderRevCorrector

Input:
- S (the $m \times n$ stoichiometric matrix)
- $Irr \subseteq \{1, \ldots, n\}$ (the set of irreversible reactions)

Output:
- $Blk^\star$ (the set of blocked reactions)
- $S^\star$ (the stoichiometric matrix after correcting the reversibility of reactions)
- $Irr^\star$ (the set of irreversible reactions after correcting the reversibility of reactions)

```
Initialization:
Blk* := ∅;
Irr* := Irr;
S* := S;

foreach i ∈ {1, ..., n} do
    Con := {Sv = 0, v_r ≥ 0 for all r ∈ Irr};
    max := maximize(v_i | Con);
    if i ∈ Rev then
        min := minimize(v_i | Con);
        if min = max = 0 then
            Blk* = Blk* ∪ {i};
            S := [S_{[m],[i−1]},  0,  S_{[m],{i+1,...,n}}];     ▷replace the i-th column with zero vector
        end
        else if min = 0, max > 0 then
            Irr* := Irr* ∪ {i};
        end
        else if min < 0, max = 0 then
            Irr* := Irr* ∪ {i};
            S := [S_{[m],[i−1]},  −S_{[m],{i}},  S_{[m],{i+1,...,n}}];     ▷multiply by −1 the i-th column
        end
    else
        if max = 0 then
            Blk* = Blk* ∪ {i};
            S := [S_{[m],[i−1]},  0,  S_{[m],{i+1,...,n}}];     ▷replace the i-th column with zero vector
        end
    end
end
```

EFP-FCA is a recent FCA method suggested by Kaleta et al. [5]. In EFP-FCA we first split the reversible reactions. Subsequently, we use the standard implementation of the EFPTools package [6] (which uses CLP to solve MILPs) for computing EFPs [5]. Then directional and partial coupling, and also uncoupling relations are inferred. Since full and partial coupling relations are not distinguishable, additional LPs have to be solved. The whole procedure is summarized in Algorithm 4. A new function, ComputeElementaryFluxPatterns$(v, S^\star)$ is used, which computes the EFPs of a network (with stoichiometric matrix $S^\star$) for a subnetwork of selected reactions in v.

Algorithm 4: EFP-FCA, an FCA approach based on elementary flux patterns [5]

Input:
- S (the $m \times n$ stoichiometric matrix)
- $Irr \subseteq \{1, \ldots, n\}$ (the set of irreversible reactions)

Output:
- Blk (the set of blocked reactions)
- $CouplingRelation$

Preprocessing:

```
/* Finding blocked reactions and updating Irr and splitting reversible reactions */
```

$(Blk^\star, S^\star, Irr^\star) := \textsc{BlkFinderRevCorrector}(S, Irr)$;

$Rev^\star := [n] \setminus (Irr^\star \cup Blk^\star)$;

$S' := \left[S,\ -S_{[m],Rev}\right]$;

Main procedure:

foreach $i, j \in [n + |Rev^\star|] \setminus Blk^\star$ *with* $i < j$ **do**
  $S'' := S'$;
  if $j \in Rev^\star, j \leq n$ **then** replace the $(j+n)$-th column of S'' with zero column;
  if $j \in Rev^\star, j \geq n+1$ **then** replace the $(j-n)$-th column of S'' with zero column;
  if $i \in Rev^\star, i \leq n$ **then** replace the $(i+n)$-th column of S'' with zero column;
  if $i \in Rev^\star, i \geq n+1$ **then** replace the $(i-n)$-th column of S'' with zero column;
  $E := \textsc{ComputeElementaryFluxPatterns}((i,j), S'')$;
  switch E **do**
    case $\{(1,1)\}$
      $CouplingRelation[i,j] :=$ "$\longleftrightarrow$";
      $min := minimize(v_i \mid \{S''v = 0, v \geq 0, v_j = 1\})$;
      $max := maximize(v_i \mid \{S''v = 0, v \geq 0, v_j = 1\})$;
      if $min = max$ **then** $CouplingRelation[i,j] :=$ "$\Longleftrightarrow$";
    case $\{(1,1),(0,1)\}$
      $CouplingRelation[i,j] :=$ "$\longrightarrow$";
    case $\{(1,1),(1,0)\}$
      $CouplingRelation[i,j] :=$ "$\longleftarrow$";
    case $\{(1,0),(0,1)\}$
      $CouplingRelation[i,j] :=$ "$\overset{Un}{\longleftrightarrow}$";
    end
  end
end

Postprocessing:

for *every* i, j *with* $i \in Rev^\star$ *and* $j \in Irr^\star$ **do**
  ⓐ $:= CouplingRelation[i,j]$;
  ⓑ $:= CouplingRelation(i+n, j)$;
  $CouplingRelation[i,j] := \textsc{CouplingRelationRecompute}$(ⓐ, ⓑ);
end

for *every* $i, j \in Rev^\star$ *with* $i < j$ **do**
  ⓐ $:= CouplingRelation[i,j]$;
  ⓑ $:= CouplingRelation(i+n, j)$;
  ⓝ $:= \textsc{CouplingRelationRecompute}$(ⓐ, ⓑ);

  ⓐ $:= CouplingRelation(i, j+n)$;
  ⓑ $:= CouplingRelation(i+n, j+n)$;
  ⓟ $:= \textsc{CouplingRelationRecompute}$(ⓐ, ⓑ);

  $CouplingRelation[i,j] := \textsc{CouplingRelationRecompute}$(ⓝ, ⓟ);
end

Implementation of FCF

FCF is based on linear programming (LP) [7]. For every pair of irreversible reactions, two LPs are solved, in which the flux through one reaction is fixed and the flux through the other reaction is maximized or minimized. Based on the optimal values, the flux coupling relation between these two reactions can be inferred. After computing all coupling relations, a post-processing step is required to obtain the coupling relations for the fluxes in the original network. Algorithm 5 summarizes the procedure.

Algorithm 5: FCF, the classical Flux Coupling Finder algorithm [7]

Input:
- S (the $m \times n$ stoichiometric matrix)
- $Irr \subseteq \{1, \ldots, n\}$ (the set of irreversible reactions)

Output:
- Blk (the set of blocked reactions)
- $CouplingRelation$

Preprocessing:

```
/* Finding blocked reactions and updating Irr and splitting reversible reactions */
```
$(Blk^\star, S^\star, Irr^\star) :=$ BlkFinderRevCorrector(S, Irr);
$Rev^\star := [n] \setminus (Irr^\star \cup Blk^\star)$;
$S' := \left[S,\ -S_{[m],Rev}\right]$;

Main procedure:

foreach $i, j \in [n + |Rev^\star|] \setminus Blk^\star$ **do**
  $S'' := S'$;
  if $j \in Rev^\star, j \leq n$ **then** replace the $(j+n)$-th column of S'' with zero column;
  if $j \in Rev^\star, j \geq n+1$ **then** replace the $(j-n)$-th column of S'' with zero column;
  if $i \in Rev^\star, i \leq n$ **then** replace the $(i+n)$-th column of S'' with zero column;
  if $i \in Rev^\star, i \geq n+1$ **then** replace the $(i-n)$-th column of S'' with zero column;
  $min := minimize(v_i \mid \{S''v = 0, v \geq 0, v_j = 1\})$; $max := maximize(v_i \mid \{S''v = 0, v \geq 0, v_j = 1\})$;
end
switch (min, max) **do**
  case $(0, \infty)$
    $CouplingRelation[i,j] :=$ "$\overset{Un}{\longleftrightarrow}$";
  case $(>0, \infty)$
    $CouplingRelation[i,j] :=$ "$\longleftarrow$";
  case $(0, >0)$
    $CouplingRelation[i,j] :=$ "$\longrightarrow$";
  case $(>0, >0)$
    if $(min = max)$ **then**
      $CouplingRelation[i,j] :=$ "$\Longleftrightarrow$";
    else
      $CouplingRelation[i,j] :=$ "$\longleftrightarrow$";
    end
  end
end

(continued on next page)

Algorithm 5: FCF, the classical Flux Coupling Finder algorithm, continued

Postprocessing:

for *every* i, j *with* $i \in Rev^\star$ *and* $j \in Irr^\star$ **do**
  ⓐ $:= CouplingRelation[i, j]$;
  ⓑ $:= CouplingRelation[i + n, j]$;
  $CouplingRelation[i, j] :=$ CouplingRelationRecompute(ⓐ, ⓑ);
end
for *every* $i, j \in Rev^\star$ *with* $i < j$ **do**
  ⓐ $:= CouplingRelation[i, j]$;
  ⓑ $:= CouplingRelation[i + n, j]$;
  ⓝ := CouplingRelationRecompute(ⓐ, ⓑ);
  ⓐ $:= CouplingRelation[i, j + n]$;
  ⓑ $:= CouplingRelation[i + n, j + n]$;
  ⓟ := CouplingRelationRecompute(ⓐ, ⓑ);
  $CouplingRelation[i, j] :=$ CouplingRelationRecompute(ⓝ, ⓟ);
end

Implementation of W-FCF

W-FCF and WR-FCF are two alternative implementations of the FCF algorithm, in which splitting of reversible reactions is not necessary. Splitting reactions slows down the algorithm for a variety of reasons, including the bigger size of the reconfigured network, the larger number of LPs to be solved, and the need for post-processing. W-FCF (and also WR-FCF) has been implemented as splitting-free FCF method to get a fair comparison between our feasibility-based FFCA and the optimality-based FCF.

W-FCF uses a new function RevToIrrevCoupling to compute the flux coupling relations between a reversible and an irreversible reaction. This function reads the maximum and minimum flux value through a reversible reaction, when the flux through the irreversible reaction is set to a constant. The output of the function is the flux coupling relation between the two reactions. It should be noted that the same function can be used for computing the coupling relation between a pair of reversible reactions i and j. First the coupling relation between i and j^+, and also between i and j^-, are computed as explained above. Then, CouplingRelationRecompute can be used to obtain the flux coupling relation between i and j.

Algorithm 6: The RevToIrrevCoupling procedure

Input:
- *min* (the minimum value of the reversible flux when the irreversible flux is constant)
- *max* (the maximum value of the reversible flux when the irreversible flux is constant)

Output:
- ⓡ (the coupling relation between the reversible and the irreversible flux)

```
switch (min, max) do
    case (−∞, < 0)
        ⓡ := "⟵";
    case (−∞, ≥ 0)
        ⓡ := "⟷ (Un)";
    case (> 0, > 0)
        if (min = max) then
            ⓡ := "⟺";
        else
            ⓡ := "⟷";
        end
    case (< 0, < 0)
        if (min = max) then
            ⓡ := "⟺";
        else
            ⓡ := "⟷";
        end
    case (≤ 0, ≥ 0)
        ⓡ := "⟶";
    case (< 0, ∞)
        ⓡ := "⟷ (Un)";
    case (−∞, ∞)
        ⓡ := "⟷ (Un)";
    case (> 0, ∞)
        ⓡ := "⟵";
    end
end
```

To find flux coupling relations between a pair of reactions, W-FCF considers three cases (in contrast to the classical FCF algorithm). If both reactions are irreversible, the procedure is similar to the FCF method. However, if one reaction is reversible and the other is irreversible, the new RevToIrrevCoupling function is used. Finally, when both reactions are reversible, the coupling relations between one reaction and the forward or backward direction of the other is computed with RevToIrrevCoupling. The final coupling relation is inferred by CouplingRelationRecompute. Algorithm 7 summarizes the procedure.

Algorithm 7: W-FCF, modified FCF algorithm without splitting reactions

Input:
- S (the $m \times n$ stoichiometric matrix)
- $Irr \subseteq \{1, \ldots, n\}$ (the set of irreversible reactions)

Output:
- Blk (the set of blocked reactions)
- $CouplingRelation$

Preprocessing:

```
/* Finding blocked reactions and updating Irr */
(Blk*, S*, Irr*) := BlkFinderRevCorrector(S, Irr);
Rev* := [n] \ (Irr* ∪ Blk*);
```

Main procedure:

```
foreach i, j ∈ Irr* with i < j do
    min := minimize(v_i | {S*v = 0, v_j = 1, v_r ≥ 0 for all r ∈ Irr*});
    max := maximize(v_i | {S*v = 0, v_j = 1, v_r ≥ 0 for all r ∈ Irr*});
    switch (min, max) do
        case (0, ∞)
            CouplingRelation[i, j] := "<--Un-->";
        case (> 0, ∞)
            CouplingRelation[i, j] := "<--";
        case (0, > 0)
            CouplingRelation[i, j] := "-->";
        case (> 0, > 0)
            if (min = max) then
                CouplingRelation[i, j] := "<==>";
            else
                CouplingRelation[i, j] := "<-->";
            end
        end
    end
end
foreach i ∈ Irr* and j ∈ Rev* do
    min := minimize(v_i | {S*v = 0, v_j = 1, v_r ≥ 0 for all r ∈ Irr*});
    max := maximize(v_i | {S*v = 0, v_j = 1, v_r ≥ 0 for all r ∈ Irr*});
    CouplingRelation[i, j] := RevToIrrevCoupling(min, max);
end
foreach i, j ∈ Rev* with i < j do
    min_1 := minimize(v_i | {S*v = 0, v_j = 1, v_r ≥ 0 for all r ∈ Irr*});
    max_1 := maximize(v_i | {S*v = 0, v_j = 1, v_r ≥ 0 for all r ∈ Irr*});
    min_2 := minimize(v_i | {S*v = 0, v_j = −1, v_r ≥ 0 for all r ∈ Irr*});
    max_2 := maximize(v_i | {S*v = 0, v_j = −1, v_r ≥ 0 for all r ∈ Irr*});
    ⓐ := RevToIrrevCoupling(min_1, max_1) ⓑ := RevToIrrevCoupling(min_2, max_2)
    CouplingRelation[i, j] := CouplingRelationRecompute(ⓐ, ⓑ)
end
```

Implementation of WR-FCF

WR-FCF is a second improved version of the FCF algorithm. As before, reversible reactions are not split. In addition, the number of LPs to be solved is reduced by applying the Reversibility-Type prunings [3]. Algorithm 8 summarizes the procedure. The function SetToOrderedVec(A) receives a set of numbers and transforms it into an ordered vector. For example, SetToOrderedVec($\{5, 1, 6, 3\}$) = $(1, 3, 5, 6)$.

Algorithm 8: WR-FCF, modified FCF algorithm without splitting reactions and with RT-prunings

```
Input:
  • S (the m × n stoichiometric matrix)
  • Irr ⊆ {1, ..., n} (the set of irreversible reactions)
Output:
  • Blk (the set of blocked reactions)
  • CouplingRelation
Preprocessing:
/* Finding blocked reactions and updating Irr */
(Blk*, S*, Irr*) := BlkFinderRevCorrector(S, Irr);
Rev* := [n] \ (Irr* ∪ Blk*); ω := SetToOrderedVec(Rev*);

/* Classification of reactions based on their reversibility types */
Irev := Irr*;
K = NullSpace(S_{[m],Rev*}); c := NumberOfColumns(K);
foreach i ∈ Rev* do
    if K_{{i},[c]} = 0^T then
        Prev := Prev ∪ {ω_i};
    else Frev := Frev ∪ {ω_i};
end
Main Procedure:
foreach i, j ∈ Irev with i < j do
    min := minimize(v_i | {S*v = 0, v_j = 1, v_r ≥ 0 for all r ∈ Irr*});
    max := maximize(v_i | {S*v = 0, v_j = 1, v_r ≥ 0 for all r ∈ Irr*});
    switch (min, max) do
        case (0, ∞)
            CouplingRelation[i, j] := "<--Un-->";
        case (> 0, ∞)
            CouplingRelation[i, j] := "<--";
        case (0, > 0)
            CouplingRelation[i, j] := "-->";
        case (> 0, > 0)
            if (min = max) then
                CouplingRelation[i, j] := "<==>";
            else
                CouplingRelation[i, j] := "<-->";
            end
        end
    end
end
```

(continued on next page)

Algorithm 8: WR-FCF, modified FCF algorithm without splitting reactions and with RT-prunings, continued

foreach $i \in Irev$ *and* $j \in Prev$ **do**
$min := minimize(v_i \mid \{S^\star v = 0, v_j = 1, v_r \geq 0 \text{ for all } r \in Irr^\star\})$;
$max := maximize(v_i \mid \{S^\star v = 0, v_j = 1, v_r \geq 0 \text{ for all } r \in Irr^\star\})$;
$CouplingRelation[i, j] := \textsc{RevToIrrevCoupling}(min, max)$;
end
foreach $i, j \in Prev$ *or* $i, j \in Frev$*, with* $i < j$ **do**
$min_1 := minimize(v_i \mid \{S^\star v = 0, v_j = 1, v_r \geq 0 \text{ for all } r \in Irr^\star\})$;
$max_1 := maximize(v_i \mid \{S^\star v = 0, v_j = 1, v_r \geq 0 \text{ for all } r \in Irr^\star\})$;
$min_2 := minimize(v_i \mid \{S^\star v = 0, v_j = -1, v_r \geq 0 \text{ for all } r \in Irr^\star\})$;
$max_2 := maximize(v_i \mid \{S^\star v = 0, v_j = -1, v_r \geq 0 \text{ for all } r \in Irr^\star\})$;
ⓐ := $\textsc{RevToIrrevCoupling}(min_1, max_1)$;
ⓑ := $\textsc{RevToIrrevCoupling}(min_2, max_2)$;
$CouplingRelation[i, j] := \textsc{CouplingRelationRecompute}$(ⓐ, ⓑ);
end

Implementation of WRP-FCF

WRP-FCF (Algorithm 9) is a third improved version of the FCF algorithm based on results in [8]. In addition to the previous optimizations in WR-FCF, it includes the PF-improvement.

Algorithm 9: WRP-FCF, modified FCF algorithm without splitting reactions, with RT-prunings and PF-improvement [8]

Input:
- S (the $m \times n$ stoichiometric matrix)
- $Irr \subseteq \{1, \ldots, n\}$ (the set of irreversible reactions)

Output:
- Blk (the set of blocked reactions)
- $CouplingRelation$

Preprocessing:
`/* Finding blocked reactions and updating` Irr `*/`
$(Blk^\star, S^\star, Irr^\star) := \textsc{BlkFinderRevCorrector}(S, Irr)$;
$Rev^\star := [n] \setminus (Irr^\star \cup Blk^\star)$; $\omega := \textsc{SetToOrderedVec}(Rev^\star)$;

`/* Classification of reactions based on their reversibility types */`
$Irev := Irr^\star$;
$K = \textsc{NullSpace}(S_{[m],Rev^\star})$; $c := \textsc{NumberOfColumns}(K)$;
foreach $i \in Rev^\star$ **do**
if $K_{\{i\},[c]} = 0^T$ **then**
$Prev := Prev \cup \{\omega_i\}$;
else $Frev := Frev \cup \{\omega_i\}$;
end

(continued on next page)

Algorithm 9: WRP-FCF, modified FCF algorithm without splitting reactions, with RT-prunings and PF-improvement, continued

Main Procedure:

foreach $i, j \in Irev$ *with* $i < j$ **do**
  $min := minimize(v_i \mid \{S^\star v = 0, v_j = 1, v_r \geq 0 \text{ for all } r \in Irr^\star\})$;
  $max := maximize(v_i \mid \{S^\star v = 0, v_j = 1, v_r \geq 0 \text{ for all } r \in Irr^\star\})$;
  switch (min, max) **do**
    case $(0, \infty)$
      $CouplingRelation[i, j] :=$ "$\overset{Un}{\longleftrightarrow}$";
    case $(> 0, \infty)$
      $CouplingRelation[i, j] :=$ "$\longleftarrow$";
    case $(0, > 0)$
      $CouplingRelation[i, j] :=$ "$\longrightarrow$";
    case $(> 0, > 0)$
      if $(min = max)$ **then**
        $CouplingRelation[i, j] :=$ "$\Longleftrightarrow$";
      else
        $CouplingRelation[i, j] :=$ "$\longleftrightarrow$";
      end
    end
  end
end
foreach $i \in Irev$ *and* $j \in Prev$ **do**
  $min := minimize(v_i \mid \{S^\star v = 0, v_j = 1, v_r \geq 0 \text{ for all } r \in Irr^\star\})$;
  $max := maximize(v_i \mid \{S^\star v = 0, v_j = 1, v_r \geq 0 \text{ for all } r \in Irr^\star\})$;
  $CouplingRelation[i, j] :=$ RevToIrrevCoupling(min, max);
end
foreach $i, j \in Prev$ *or* $i, j \in Frev$, *with* $i < j$ **do**
  $m := maximize(v_i \mid \{S^\star v = 0, v_j = 0, 0 \leq v_i \leq 1\})$;
  if $m = 0$ **then**
    $CouplingRelation[i, j] :=$ "$\Longleftrightarrow$";
  else
    $CouplingRelation[i, j] :=$ "$\overset{Un}{\longleftrightarrow}$";
  end
end

Implementation of FFCA

In FFCA (Feasibility-based Flux Coupling Analysis), we first classify the reactions into different subsets based on their reversibility types [3]. Based on this classification, the feasibility of two LPs is checked in those cases where flux coupling is possible (see the Results and Discussion section in the main paper). As in the FCF implementation, we use the CLP for solving LPs. Algorithm 10 summarizes the procedure.

Algorithm 10: FFCA, Feasibility-based flux coupling analysis

Input:
- S (the $m \times n$ stoichiometric matrix)
- $Irr \subseteq \{1, \ldots, n\}$ (the set of irreversible reactions)

Output:
- Blk (the set of blocked reactions)
- $CouplingRelation$

Initialization:
$Blk := \emptyset; Irev := \emptyset; Prev := \emptyset; Frev := \emptyset; Rev^* := \emptyset;$

Preprocessing:
`/* Finding blocked reactions and updating` Irr `*/`
$(Blk^\star, S^\star, Irr^\star) := \textsc{BlkFinderRevCorrector}(S, Irr);$
$Rev^\star := [n] \setminus (Irr^\star \cup Blk^\star)$; $\omega := \textsc{SetToOrderedVec}(Rev^\star);$

`/* Classification of reactions based on their reversibility types */`
$Irev := Irr^\star;$
$K = \textsc{NullSpace}(S_{[m],Rev^\star})$; $c := \textsc{NumberOfColumns}(K);$
foreach $i \in Rev^\star$ **do**
  if $K_{\{i\},[c]} = 0^T$ **then**
    $Prev := Prev \cup \{\omega_i\};$
  else $Frev := Frev \cup \{\omega_i\};$
end

Main procedure:
foreach $i, j \in Irev$ *with* $i < j$ **do**
  $m_1 := maximize(0 \mid \{v_i = 1, v_j = 0, S^\star v = 0, v_r \geq 0 \text{ for all } r \in Irr^\star\});$
  $m_2 := maximize(0 \mid \{v_i = 0, v_j = 1, S^\star v = 0, v_r \geq 0 \text{ for all } r \in Irr^\star\});$
  if $m_1, m_2 \in \mathbb{R}$ **then**
    $CouplingRelation[i, j] :=$ "$\overset{Un}{\longleftrightarrow}$";
  else if $m_1 \in \mathbb{R}$ *and* $m_2 = \perp$ **then**
    $CouplingRelation[i, j] :=$ "$\longleftarrow$";
  else if $m_1 = \perp$ *and* $m_2 \in \mathbb{R}$ **then**
    $CouplingRelation[i, j] :=$ "$\longrightarrow$";
  else
    $CouplingRelation[i, j] :=$ "$\longleftrightarrow$";
    $min := minimize(v_i \mid \{v_j = 1, S^\star v = 0, v_r \geq 0 \text{ for all } r \in Irr^\star\});$
    $max := maximize(v_i \mid \{v_j = 1, S^\star v = 0, v_r \geq 0 \text{ for all } r \in Irr^\star\});$
    if $min = max$ **then** $CouplingRelation[i, j] :=$ "$\Longleftrightarrow$";
  end
end
foreach $i \in Prev$ *and* $j \in Irev$ **do**
  $m_1 := maximize(0 \mid \{v_i = 1, v_j = 0, S^\star v = 0, v_r \geq 0 \text{ for all } r \in Irr^\star\});$
  $m_2 := maximize(0 \mid \{v_i = -1, v_j = 0, S^\star v = 0, v_r \geq 0 \text{ for all } r \in Irr^\star\});$
  if $m_1 = \perp$ *and* $m_2 = \perp$ **then**
    $CouplingRelation[i, j] :=$ "$\longrightarrow$";
  else
    $CouplingRelation[i, j] :=$ "$\overset{Un}{\longleftrightarrow}$";
  end
end

(continued on next page)

Algorithm 10: FFCA, Feasibility-based flux coupling analysis, continued

foreach $i, j \in Prev$ *or* $i, j \in Frev$ **do**
  $m_1 := maximize(0 \mid \{v_i = 1, v_j = 0, S^\star v = 0\})$;
  if $m_1 = \perp$ **then**
    $CouplingRelation[i, j] :=$ "$\Longleftrightarrow$";
  else
    $CouplingRelation[i, j] :=$ "$\overset{Un}{\longleftrightarrow}$";
  end
end

References

1. Fukuda K, Prodon A: **Double description method revisited**. In *Combinatorics and Computer Science: 8th Franco-Japanese and 4th Franco-Chinese Conference. Brest, France*, *Volume 1120 of* Lecture Notes in Computer Science 1996:91–111.
2. Larhlimi A, Bockmayr A: **A new constraint-based description of the steady-state flux cone of metabolic networks**. *Discrete Applied Mathematics* 2009, **157**:2257–2266.
3. Larhlimi A, Bockmayr A: **A new approach to flux coupling analysis of metabolic networks**. In *Computational Life Sciences II, Second International Symposium (CompLife 2006), Cambridge, UK*, *Volume 4216 of* Lecture Notes in Computer Science 2006:205–215.
4. Lougee-Heimer R: **The Common Optimization INterface for Operations Research: Promoting open-source software in the operations research community**. *IBM Journal of Research and Development* 2003, **47**:57–66.
5. Kaleta C, de Figueiredo LF, Schuster S: **Can the whole be less than the sum of its parts? Pathway analysis in genome-scale metabolic networks using elementary flux patterns.** *Genome Research* 2009, **19**:1872–1883.
6. Kaleta C: **EFPTools, for computing elementary flux patterns (EFPs)** [http://users.minet.uni-jena.de/~m3kach/EFPA/].
7. Burgard AP, Nikolaev EV, Schilling CH, Maranas CD: **Flux coupling analysis of genome-scale metabolic network reconstructions**. *Genome Research* 2004, **14**:301–312.
8. Larhlimi A: **New concepts and tools in constraint-based analysis of metabolic networks**. *PhD thesis*, Freie Universität Berlin 2008, [http://www.diss.fu-berlin.de/diss/receive/FUDISS_thesis_000000009198].
