# Supplementary material for: FFCA: a feasibility-based method for flux coupling analysis of metabolic networks
Source: BMC Bioinformatics. 2011 Jun 15;12:236. doi: 10.1186/1471-2105-12-236 (PMC3144024; doi:10.1186/1471-2105-12-236)
Supplement: Additional file 2 — Dependence of flux coupling analysis on the number of reversible reactions. In this file, we show how flux coupling relations depend on the number of reversible reactions in the E. coli metabolic network. [file 1471-2105-12-236-S2.PDF]

Additional file 2: Dependence of flux coupling analysis on the number of reversible reactions

Since FFCA includes the RT-prunings and the PF-improvement, it might be interesting to see how the number of flux coupling relations and also the running time of FFCA depend on the number of reversible reactions in a network.

The metabolic network of *E. coli* (iJR904 model) [1] is one of the networks that we use for this study. Based on this model, Kümmel et al. [2] introduced a modified version where the reversibility types of some reactions were changed. Most of these changes were from “irreversible” to “reversible” (from iJR904 to the modified network). In order to study how the number of irreversible reactions in a network influences flux coupling analysis, we performed the following computational experiment:

(1) From Ref. [2], Additional file 1 was downloaded as an Excel file. The list of reactions in the first sheet was considered. This model is called the “Min-Irr” model, which means the model with “the minimal set of irreversible reactions”.

(2) From the first sheet, the reaction IDs were extracted and compared to the reaction IDs in the *E. coli* model iJR904. In case of 14 reaction IDs, there was no match. Therefore, we detected the matching reactions manually, by comparing the metabolites involved in each reaction.

(3) The reversibility types of the reactions in “Min-Irr” were compared to the reversibility types of reactions in iJR904. We found a set (denoted by R) of 70 irreversible reactions in iJR904 which are reversible in “Min-Irr”. Additionally, 6 reversible reactions in iJR904 were found to be irreversible in “Min-Irr”. To simplify the experiment, we decided to ignore these 6 reactions. Therefore, a metabolic model M1 was constructed which was the same as iJR904 except for a random subset of R containing 7 reactions, S7, which were assumed to be reversible in M1. Similarly, other models M2, ..., M10 were generated by selecting subsets S14, …, S70, where S7⊂S14⊂…⊂S70.

The running times and also the detailed information about flux coupling relations are presented in Table A1 (for the 765 unblocked reactions in all of the cases):

	full	partial	directional	uncoupled	running time (sec)
**iJR904**	2567	68	6208	283387	5.7e+3
**M1**	2567	68	6000	283595	4.8e+3
**M2**	2567	68	6000	283595	4.7e+3
**M3**	2567	68	5994	283601	4.5e+3
**M4**	2567	68	5984	283611	4.3e+3
**M5**	2567	68	5984	283611	4.2e+3
**M6**	2567	68	5984	283611	4.2e+3
**M7**	2567	68	5937	283658	4.1e+3
**M8**	2567	68	5937	283658	3.9e+3
**M9**	2567	68	5937	283658	3.9e+3
**M10**	2567	68	5937	283658	3.9e+3

Table A1: Flux coupling relations and the running time of FFCA tool as a function of the number of reversible reactions in the network

As expected, the numbers of uncoupled pairs generally increase (and the running times generally decrease) with the increase in the number of reversible reactions. Note that for the FCA approaches with splitting reversible reactions (e.g. FCF), the running times are expected to increase with the number of reversible reactions in the network, as splitting the reversible reactions increases the number of linear programs that have to be solved.

References:

1. Reed JL, Vo TD, Schilling CH, Palsson BO: **An expanded genome-scale model of *Escherichia coli* K-12 (iJR904 GSM/GPR)**. *Genome Biology* 2003, **4**:R54.

2. Kümmel A, Panke S, Heinemann M: **Systematic assignment of thermodynamic constraints in metabolic network models**. *BMC Bioinformatics* 2006, **7**:512.
